# Association of Anthracycline With Heart Failure in Patients Treated for Breast Cancer or Lymphoma, 1985-2010

**DOI:** 10.1001/jamanetworkopen.2022.54669

**Published:** 2023-02-03

**Authors:** Carolyn M. Larsen, Mariana Garcia Arango, Harika Dasari, Maria Arciniegas Calle, Effie Adjei, Juan Rico Mesa, Christopher G. Scott, Carrie A. Thompson, James R. Cerhan, Tufia C. Haddad, Matthew P. Goetz, Joerg Herrmann, Hector R. Villarraga

**Affiliations:** 1 Department of Cardiovascular Medicine, Mayo Clinic, Scottsdale, Arizona; 2 Department of Cardiovascular Medicine, Mayo Clinic, Rochester, Minnesota; 3 Division of Pulmonary and Critical Care Medicine, Mayo Clinic, Rochester, Minnesota; 4 Department of Quantitative Health Sciences, Mayo Clinic, Rochester, Minnesota; 5 Division of Hematology, Mayo Clinic, Rochester, Minnesota; 6 Division of Medical Oncology, Mayo Clinic, Rochester, Minnesota

## Abstract

**Importance:**

Anthracyclines increase the risk for congestive heart failure (CHF); however, long-term cumulative incidence and risk factors for CHF after anthracycline therapy are not well defined in population-based studies.

**Objective:**

To compare the long-term cumulative incidence of CHF in patients with breast cancer or lymphoma treated with anthracycline therapy compared with healthy controls from the same community.

**Design, Setting, and Participants:**

This retrospective population-based case-control study included data from the Rochester Epidemiology Project. Participants included residents of Olmsted County, Minnesota, diagnosed with breast cancer or lymphoma from January 1985 through December 2010 matched for age, sex, and comorbidities with healthy controls, with a final ratio of 1 case to 1.5 controls. Statistical analysis was performed between July 2017 and February 2022.

**Exposures:**

Cancer treatment and CHF risk factors.

**Main Outcomes and Measures:**

The main outcome was new-onset CHF, as defined by the modified Framingham criteria. Cox proportional hazards regression was used to estimate hazard ratios (HRs) to compare the risk of CHF in participants with cancer vs controls, adjusted for age, sex, diabetes, hypertension, hyperlipidemia, coronary artery disease, obesity, and smoking history.

**Results:**

A total of 2196 individuals were included, with 812 patients with cancer and 1384 participants without cancer. The mean (SD) age was 52.62 (14.56) years and 1704 participants (78%) were female. Median (IQR) follow-up was 8.6 (5.2-13.4) years in the case group vs 12.5 (8.7-17.5) years in the control group. Overall, patients with cancer had higher risk of CHF compared with the control cohort even after adjusting for age, sex, diabetes, hypertension, coronary artery disease, hyperlipidemia, obesity, and smoking status (HR, 2.86 [95% CI, 1.90-4.32]; *P* < .001). After adjusting for the same variables, CHF risk was greater for patients with cancer receiving anthracycline (HR, 3.25 [95% CI, 2.11-5.00]; *P* < .001) and was attenuated and lost statistical significance for patients with cancer not receiving anthracyclines (HR, 1.78 [95% CI, 0.83-3.81]; *P* = .14). Higher cumulative incidence for patients treated with anthracyclines vs comparator cohort was observed at 1 year (1.81% vs 0.09%), 5 years (2.91% vs 0.79%), 10 years (5.36% vs 1.74%), 15 years (7.42% vs 3.18%), and 20 years (10.75% vs 4.98%) (*P* < .001). There were no significant differences in risk of CHF for patients receiving anthracycline at a dose of less than 180 mg/m^2^ compared with those at a dose of 180 to 250 mg/m^2^ (HR, 0.54 [95% CI, 0.19-1.51]) or at a dose of more than 250 mg/m^2^ (HR, 1.23 [95% CI, 0.52-2.91]). At diagnosis, age was an independent risk factor associated with CHF (HR per 10 years, 2.77 [95% CI, 1.99-3.86]; *P* < .001).

**Conclusions and Relevance:**

In this retrospective population-based case-control study, anthracyclines were associated with an increased risk of CHF early during follow-up, and the increased risk persisted over time. The cumulative incidence of CHF in patients with breast cancer or lymphoma treated with anthracyclines at 15 years was more than 2-fold that of the control group.

## Key Points

**Question:**

What is the long-term incidence of heart failure (HF) in patients with cancer treated with anthracyclines in a population-based sample?

**Findings:**

In this case-control study among 2196 individuals, including 812 participants with cancer and 1384 control participants, the cumulative incidence of HF in participants with cancer treated with anthracycline was 7.4% over 15 years, more than 2 times higher than in matched controls, and was not significant for a subgroup of participants with cancer not treated with anthracycline compared with controls. The shift in risk was observed during the first year of follow-up and persisted over the follow-up period.

**Meaning:**

These findings suggest that anthracycline treatment in patients with cancer was associated with more than twice the risk of HF compared with controls.

## Teaser

This case-control study compares the long-term incidence of congestive heart failure in patients treated for breast cancer or lymphoma with anthracycline therapy compared with healthy controls from the same community.

## Introduction

There has been more awareness of the complications associated with chemotherapeutic agents within the last decades, and it is well established that individuals who have survived cancer are at increased risk for cardiovascular disease.^1,2,3,4,5^ Anthracyclines are a class of chemotherapeutic agents used for multiple types of cancer, particularly in the frontline for breast cancer and lymphoma. They are associated with dose-dependent cardiac toxic effects, CHF, and negative impacts on quality of life.^6,7,8,9,10,11,12,13,14,15^ In a 2013 meta-analysis,^16^ clinical cardiac toxic effects were reported in 6% of patients treated with an anthracycline after a median follow-up of 9 years, and subclinical cardiac toxic effects were described in 18% of patients.

Despite the established cardiovascular risk associated with anthracycline therapy, the long-term cardiac risk and magnitude remain less well defined in population-based studies. Published clinical trials have limited follow-up durations and inclusion and exclusion criteria that decrease the validity of generalizations to other populations.^9,16,17,18^ In addition, data for long-term cardiovascular outcomes traditionally have been obtained from administrative databases that define cardiac toxic effects by diagnosis codes, which provide only moderate sensitivity and moderate positive predictive value and, thus, only an estimate of the incidence of cardiac toxic effects.^6,10,16,19,20^ Additionally, databases in smaller institutions are limited to the completeness of follow up.^6,20^

Olmsted County is a community in the upper Midwest of the United States that provides a unique opportunity for epidemiologic research via the Rochester Epidemiology Project (REP). The REP is a collaboration among health care practitioners serving the Olmsted County community that creates a research infrastructure with complete data linkage of health records. The completeness of the medical record data on a defined population available through the REP makes it valuable for epidemiologic research.^21,22^ This study aimed to analyze the cumulative incidence of CHF in residents of Olmsted County who received chemotherapy for breast cancer or lymphoma (Hodgkin or non-Hodgkin) vs that of a comparison cohort without cancer matched for age, sex, and noncancer comorbidities.

## Methods

This case-control study was approved by the institutional review boards of Mayo Clinic and Olmsted Medical Center. All participants agreed to the use their data for research purposes, per Minnesota law.^23,24,25,26^ Patients at Mayo Clinic and Olmsted Medical Center who did not provide consent by Minnesota research authorization were not included in this study. This study is reported following the Strengthening the Reporting of Observational Studies in Epidemiology (STROBE) reporting guideline.

### Study Setting

The population of Olmsted County in 2014 was approximately 150 287. It was similar to the rest of Minnesota and the upper Midwest in regard to age, sex, and race and ethnicity.^27^ Mayo Clinic and Olmsted Medical Center are the 2 leading health care systems in Olmsted County, both of which have integrated health records accessible for research purposes through the REP. We conducted a retrospective analysis of cardiovascular outcomes of patients with breast cancer and lymphoma treated with chemotherapy in this population by using the REP’s resources.

### Identification of Patients With Cancer

Adult residents (age ≥18 years) of Olmsted County who were newly diagnosed with and treated for breast cancer or lymphoma with chemotherapy with or without chest or mediastinal radiotherapy from January 1, 1985, through December 31, 2010, were identified via the Mayo Clinic Cancer Registry and the REP database with the codes described in the eAppendix in Supplement 1. Participants were excluded if they had preexisting CHF at cancer diagnosis. However, participants with cardiovascular risk factors were not excluded.

### Comparison Cohort

A cohort of participants with no history of cancer served as a background comparator for CHF incidence in the study population. The comparison participants were matched to the patients with cancer on age, calendar year, sex, and baseline comorbidities (ie, coronary artery disease, diabetes, hypertension, and obesity) at the index date through the REP database. Comparator participants were required to be followed-up at least as long as participants with cancer, and the matching ratio was 1 case to 1.5 controls. Participants were excluded if they had pre-existing CHF at the index date.

### Data Extraction

Data were extracted by a combination of electronic data extraction and manual record review. Data collection was obtained from the REP regarding date of birth, sex, body weight, date of last time the patient was censored in Olmsted County, and, when applicable, date and cause of death. Race and ethnicity were self-reported and extracted from the medical records. Participants identified as American Indian, Asian, Black, White (including both Hispanic and non-Hispanic patients), or unknown (e, patients who did not provide their race or ethinicity). Additionally, a manual record review was performed for all patients with cancer to record details of cancer therapy, comorbidities, and cardiovascular outcomes. The comparison cohort baseline comorbidities and potential cardiovascular outcomes were queried electronically. Record review was subsequently performed manually to confirm the diagnosis of CHF. In addition, manual record reviews were successfully conducted for all 1384 comparator participants to assure adequate quality and to avoid misclassifying participants who developed CHF. Coronary artery disease was defined for cases and control as coronary angiography with stenosis of at least 1 artery greater than 50%, coronary revascularization, history of myocardial infarction, presence of regional wall motion abnormality on an imaging study, or a positive stress test.

### Outcomes

The primary outcome was new-onset CHF as defined by the modified Framingham criteria, both in the inpatient and outpatient setting.^28^ All cases and controls were abstracted through April 6, 2017, with censoring at time of emigration from Olmsted County.

### Statistical Analysis

Data were collected and stored in JMP Pro software version 10 (SAS Institute). Data analysis was performed in JMP Pro software version 10 and SAS software version 9.4 (SAS Institute) between July 2017 and February 2022. Two-sided *P* = .05 was used to define statistical significance. Between-group differences were assessed using the χ^2^ test for categorical variables and the *t* test for continuous variables. Cox proportional hazards regression was used to estimate hazard ratios (HRs) to compare the risk of CHF in patients with cancer vs the comparison cohort, adjusted for age, sex, diabetes, hypertension, hyperlipidemia, coronary artery disease, obesity, and smoking history. The cumulative incidence of CHF in the cancer and comparison cohorts was modeled, accounting for the competing risk of death. CHF was analyzed in the absence of recurrence; thus, patients with cancer were censored in analyses at time of recurrence. Cumulative doxorubicin dose was defined during the initial treatment period.

We also assessed risk factors for CHF in the combined group of patients with cancer and controls as well as in the subgroups of patients with cancer who received anthracyclines and those who did not. Age was analyzed as a categorical variable in decades, given the nonlinear association of age and CHF (both for patients with cancer and the comparison cohort). Multivariable Cox regression was performed to identify independent risk factors for CHF in patients with cancer in a model that included age, sex, doxorubicin isotoxic dose, chest or mediastinal radiation, diabetes, hypertension, coronary artery disease, hyperlipidemia, obesity (defined by body mass index [calculated as weight in kilograms divided by height in meters squared] of 30 or more), and ever smoking.

## Results

A total of 2196 individuals were included, with 812 patients identified from the REP as newly diagnosed with breast cancer, Hodgkin lymphoma, or non-Hodgkin lymphoma and were treated with chemotherapy with or without radiotherapy, and 1384 patients without cancer were identified as a comparison cohort. Overall, the mean (SD) age was 52.62 (14.56) years and 1704 participants (78%) were female (Table 1). The median (IQR) follow-up for participants in the case group was 8.6 (5.2-13.4) years, while for controls, it was 12.5 (8.7-17.5) years. Patients with cancer, compared with the control group, had a higher reported history of ever smoking (347 cases [43%] vs 284 controls [21%]; *P* < .001) and coronary artery disease (42 cases [5%] vs 25 controls [2%]; *P* < .001) (Table 1).

**Table 1.** Baseline Characteristics of Study Participants

Variable	Patients No. (%)		*P* value
	Control (n = 1384)	With cancer (n = 812)	
Age, y^a^			
Mean (SD)	52.60 (14.62)	52.63 (14.53)	.96
<40	231 (17)	136 (17)	>.99
40-49	404 (29)	233 (29)	
50-59	328 (24)	199 (25)	
60-69	223 (16)	129 (16)	
70-79	133 (10)	78 (10)	
≥80	65 (5)	37 (5)	
Race			
American Indian	2 (<1)	1 (<1)	.21
Asian	16 (1)	19 (2)	
Black	11 (1)	8 (1)	
White^b^	1283 (93)	750 (92)	
Unknown^c^	72 (5)	34 (4)	
Sex			
Female	1081 (78)	623 (77)	.45
Male^a^	303 (22)	189 (23)	
Diabetes^a^	93 (7)	64 (8)	.31
Hypertension^a^	370 (27)	246 (30)	.07
Index CAD >50%, MI, and stress clinical diagnoses^a^	25 (2)	42 (5)	<.001
Hyperlipidemia	533 (39)	313 (39)	.99
BMI >30^a^	432 (31)	247 (30)	.70
History of smoking	284 (21)	347 (43)	<.001

Abbreviations: BMI, body mass index (calculated as weight in kilograms divided by height in meters squared); CAD, coronary artery disease; MI, myocardial infarction.

^a^ Matching factors. Participants were age-matched by decade.

^b^ Five patients had ethnicity coded as Hispanic, Mexican, or other Spanish-speaking ethnicity and were included in White race.

^c^ Includes patients who did not provide their race or ethnicity.

### HF Incidence

**Figure 1.**
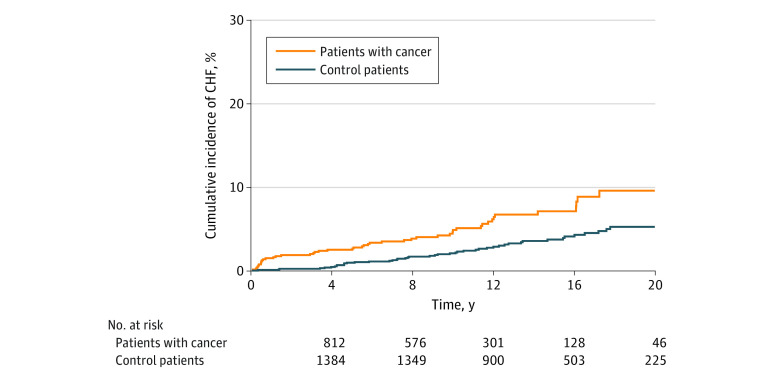
Cumulative Incidence of Congestive Heart Failure (CHF) Over Time for Patients With Cancer vs Matched Comparison Cohort Risk of CHF adjusted for age, sex, diabetes, hypertension, coronary artery disease, hyperlipidemia, obesity at baseline, and smoking history: hazard ratio, 2.86 (95% CI, 1.90-4.32); *P* < .001).

Patients with cancer had higher risk of CHF compared with the comparison cohort, even after adjusting for age, sex, diabetes, hypertension, coronary artery disease, hyperlipidemia, obesity, and smoking history (HR, 2.86 [95% CI, 1.90-4.32; *P* < .001). This risk started from the first year (12 participants with incident CHF) and persisted over time even when excluding the first-year events (adjusted HR, 2.41 [95% CI, 1.56-3.76]; *P* < .001) (Figure 1).

**Figure 2.**
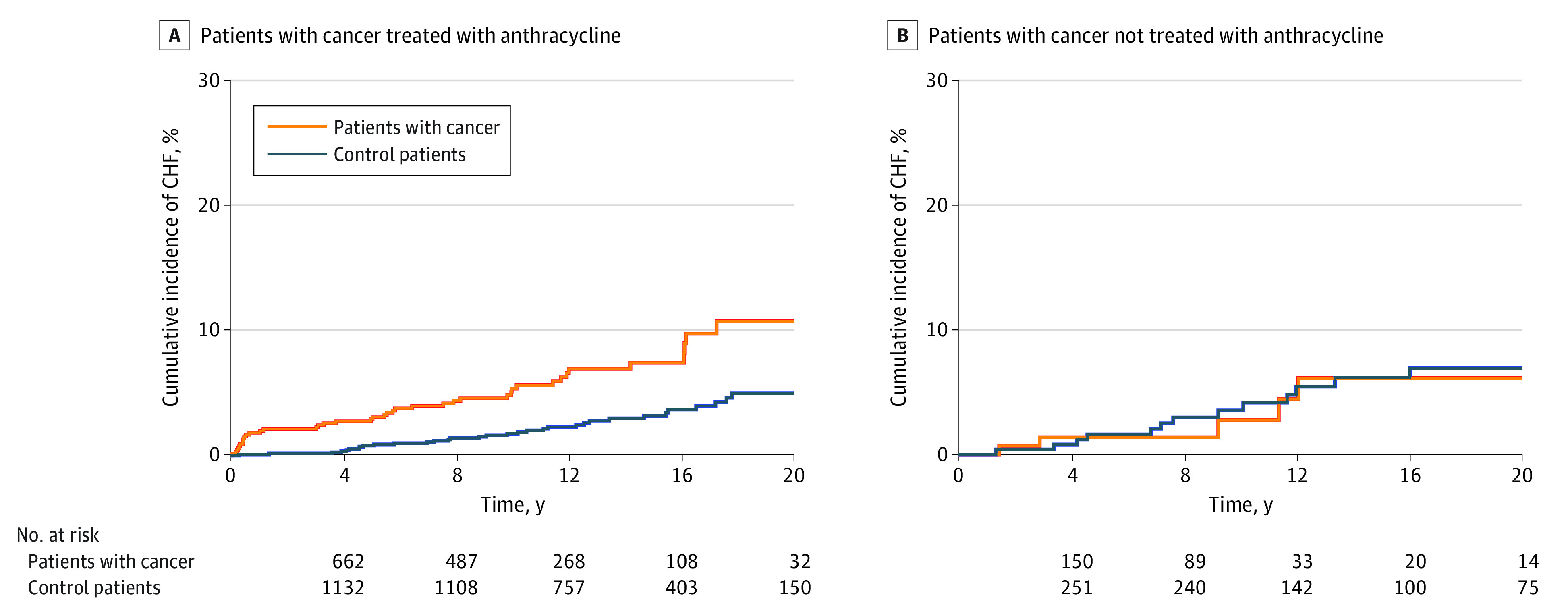
Cumulative Incidence of Congestive Heart Failure (CHF) Over Time Shown by Treatment With Anthracycline

Patients with cancer treated with anthracyclines had an elevated risk of CHF compared with the comparison cohort (HR, 3.25 [95% CI, 2.11-5.00]; *P* < .001), but this association was attenuated and lost statistical significance for patients with cancer who were not treated with anthracyclines (HR, 1.78 [95% CI, 0.83-3.81]; *P* = .14) after adjusting for the same variables. CHF cumulative incidence was significantly greater in patients with cancer treated with anthracyclines compared with the comparison cohort after 1 year (1.81% vs 0.09%), 5 years (2.91% vs 0.79%), 10 years (5.36% vs 1.74%), 15 years (7.42% vs 3.18%), 20 years (10.75% vs 4.98%), and 25 years (14.69% vs 9.02%; *P* < .001) (Figure 1). In contrast, there was no difference in the cumulative incidence of CHF between patients with cancer not treated with anthracyclines and the comparator cohort (Figure 2).

We found an increased risk of CHF associated with anthracycline treatment regardless of the dose and without evidence that risk of CHF increased with higher doses. There was no significant difference in risk of CHF for patients treated with anthracycline at a dose of less than 180 mg/m^2^ compared with patients treated with a dose of 180 to 250 mg/m^2^ (HR, 0.54 [95% CI, 0.19-1.51]) or patients treated with a dose of more than 250 mg/m^2^ (HR, 1.23 [95% CI, 0.52-2.91]). Thus, we identified that any anthracycline use was a variable associated with increased the risk of CHF (Table 2; eFigure 1 in Supplement 1).

**Table 2.** Characteristics of Patients With Cancer and Treatment Regimens

Variable	Patients, No. (%)			*P* value
	Breast cancer (n = 513)	HL (n = 71)	NHL (n = 228)	
First line chemotherapy regimen				
Anthracycline	430 (84)	68 (96)	164 (72)	<.001
Not anthracyclines	70 (14)	3 (4)	64 (28)	
Trastuzumab	13 (3)	0	0	
Radiation	343 (67)	21 (30)	4 (2)	<.001
Right breast or chest wall radiation	173 (34)	0	0	<.001
Left breast or chest wall radiation	175 (34)	1 (1)	0	<.001
Mediastinal radiation	1 (<1)	20 (28)	4 (2)	<.001
Anthracycline dose, mg/m^2^				
None^a^	83 (16)	3 (4)	64 (28)	<.001
<180	20 (4)	10 (14)	43 (19)	
180-250	370 (72)	20 (28)	22 (10)	
>250	40 (8)	38 (54)	99 (43)	
Treatment at cancer diagnosis				
β-blocker	48 (9)	3 (4)	47 (21)	<.001
ACEI or ARBs	48 (9)	4 (6)	49 (22)	<.001
Statins	50 (10)	3 (4)	38 (17)	.003

Abbreviations: ACEI, angiotensin converting enzyme inhibitor; ARB, angiotensin II receptor blocker; HL, Hodgkin lymphoma; NHL, non-Hodgkin lymphoma.

^a^ No anthracycline therapy included patients who were treated with trastuzumab.

### Risk Factors of CHF in Patients With Breast Cancer or Lymphoma Patients

When we evaluated CHF risk by type of cancer subgroups and adjusting for age, sex, diabetes, hypertension, coronary artery disease, hyperlipidemia, smoking history, and obesity, there was an increased risk of CHF for patients with breast cancer (HR 3.52 [95% CI, 1.78-6.93]; *P* < .001) or non-Hodgkin lymphoma (HR 1.99 [95% CI, 1.12-3.51]; *P* = .02). There were only 4 events in the Hodgkin lymphoma group, so we were unable to model this subgroup specifically (eFigure 2 in Supplement 1).

At diagnosis, age was an independent risk factor associated with CHF (HR per 10 years, 2.77 [95% CI, 1.99-3.86]; *P* < .001). Other comorbidities were not independently associated with an increased risk of developing CHF (Table 3).

**Table 3.** Multivariable Model of Risk Factors Associated With Congestive Heart Failure

Risk factor	Hazard Ratio (95% CI)	*P* value
Anthracycline use	2.56 (1.02-6.41)	.04
Chest or mediastinal radiation	0.32 (0.13-0.76)	.01
Age (per 10 y)	2.77 (1.99-3.86)	<.001
Male sex	0.90 (0.42-1.94)	.80
At diagnosis		
Diabetes	1.90 (0.88-4.10)	.10
Hypertension	0.90 (0.42-1.94)	.80
Coronary artery disease	1.56 (0.64-3.82)	.33
Hyperlipidemia	1.04 (0.52-2.07)	.92
BMI >30	1.25 (0.67-2.34)	.48
Ever smoker	1.60 (0.84-3.02)	.15
HL vs breast cancer	1.63 (0.37-7.25)	.52
NHL vs breast cancer	0.50 (0.20-1.27)	.15

Abbreviations: BMI, body mass index (calculated as weight in kilograms divided by height in meters squared); HL, Hodgkin lymphoma; NHL, non-Hodgkin lymphoma.

There was no difference in the risk of developing CHF among participants treated before vs after the year 2000 (HR, 0.93 [95% CI 0.44, 1.94]; *P* = .87). There was also no difference observed by baseline cardiovascular therapy using β-blockers (HR, 1.65 [95% CI, 0.73-3.74]; *P* = .23), angiotensin-converting enzyme inhibitors or angiotensin receptor blockers (HR, 1.31 [95% CI, 0.57-2.99]; *P* = .53), or statins (HR, 0.59 [95% CI, 0.23-1.52]; *P* = .27).

Use of concomitant radiation therapy (chest or mediastinal) was inversely associated with risk of CHF (HR, 0.32 [95% CI, 0.13-0.74]; *P* = .009) even when comparing left and right radiotherapy. There was no evidence of an association of concomitant radiation therapy with CHF risk by cancer type.

## Discussion

This case-control study is the first population-based study, to our knowledge, to evaluate the long-term outcome of CHF in patients with breast cancer or Hodgkin or non-Hodgkin lymphoma associated with anthracycline use compared with a comparison group from the same community. Our main findings were that patients with cancer had a nearly 3-fold greater risk of CHF than those without cancer from 1985 to 2010. This risk was greatest in patients treated with anthracyclines and was not significant for patients with cancer not treated with anthracycline compared with the comparator population.

The cumulative incidence of CHF in patients with cancer treated with anthracycline in this study was more than double that of controls at 15 years after cancer diagnosis. Our results are similar to those reported by Baech et al^29^ from a Danish nationwide cohort. Baech and colleagues^29^ compared anthracycline containing regimens with nonanthracycline in a cohort of 2440 patients with lymphoma without previous heart disease, finding that patients receiving anthracyclines had higher risks of CHF compared with patients treated without anthracyclines, for whom the overall risk was not significant.^29^

The novelty of our findings lies in comparing the risk of CHF in patients with cancer with that of a comparison cohort with similar comorbidities but without cancer, allowing a better understanding of the association of anthracyclines in the development of heart failure at the population level.

Our results contrast with those presented in a 2017 study by Salz et al^30^ in which the authors matched 2508 patients who had had non-Hodgkin lymphoma to 7399 controls by age and sex and found preexisting cardiovascular risk factors associated with the later development of CHF, contrasting with the study by Baech et al^29^ in patients who had had non-Hodgkin lymphoma without cardiovascular risk factors that found an association of CHF with the use of anthracyclines. Our study differs from the study by Salz et al^30^ in that patients were matched for age, sex, and comorbidities and demonstrates a direct association with the use of this chemotherapeutic agent.

The Pathways Heart Study^31^ from an integrated health system in northern California evaluated 13 642 patients with breast cancer and 68 202 controls matched by age and race and ethnicity and found that the 10-year cumulative incidence rate for heart failure or cardiomyopathy 4.1% among patients with breast cancer vs 2.3% among controls. Our study is population based, but we had similar findings regarding the exposure to anthracyclines in association with the incidence of CHF.

Current expert consensus recommendations^32,33^ for cardiac surveillance during and after anthracycline chemotherapy recommend a cardiac toxic effects risk evaluation approach (to categorize patients with low, medium, and high risk). The Heart Failure Association (HFA), the European Association of Cardiovascular Imaging (EACVI) and the Cardio-Oncology Council of the European Society of Cardiology (ESC) include echocardiography surveillance always at baseline, followed by echocardiographic examination after completing a cumulative dose of 250 mg/m^2^ of doxorubicin or equivalent and 12 months after treatment completion. In patients with high risk, an echocardiographic examination is recommended every 2 cycles and 3 months after treatment, as a class I indication. If higher doses of anthracyclines are administered, an echocardiographic examination should be considered every additional 50 mg/m^2^ to 100 mg/m^2^ of doxorubicin that a patient receives.^32,33^ Importantly expert guidelines suggest a 5-year reevaluation in patients with low or medium risk, and in high-risk groups, reevaluation should occur annually for 2 or 3 years after the 12-month follow-up, then in 3- to 5-year intervals. Additional data from registries and modern clinical trials are needed to assess the potential benefit associated with cardiac surveillance imaging, including evaluating the focused-risk approach highly dependent on anthracycline cumulative dose, and to define the optimal timing of such surveillance.^32,33^

Age was another independent risk factor associated with CHF in patients with cancer in our study. Our long-term follow-up data suggest that some patients with cancer treated with anthracyclines remained at increased risk of CHF decades after their cancer diagnosis. Therefore, these individuals are expected to require regular clinical follow-up to screen for and modify coexisting cardiovascular risk factors (eg, diabetes, hypertension, hyperlipidemia, body mass index, tobacco exposure, and sedentary lifestyle) and assess for early signs and symptoms of CHF.

Interestingly, radiation therapy to the chest and mediastinum did not emerge as an independent risk factor for CHF. Consistent with our study, a prospective cohort study of patients with newly diagnosed lymphoma found that patients had increased risk of cardiovascular disease, especially CHF, at 10 years, but receipt of radiation therapy was not associated with this outcome^34^ and this was also found in the study by Salz et al^30^ using a Danish registry cohort. However, not all chest radiation affects the heart to the same degree. We compared the difference between right and left radiotherapy (chest or mediastinal) in a multivariable model and found that it was not an independent risk factor. Data on the cardiac-specific radiation dose were unavailable.

The completeness and duration of follow-up for patients with cancer and our control group through the REP resources is a marked advantage. All diagnoses of CHF were able to be confirmed by a manual review of the health records. Furthermore, data regarding chemotherapeutic regimens and baseline comorbidities for all patients with were available.

### Limitations

This study has some limitations. Although there was a lack of racial and ethnic diversity in the study population, limiting the generalizability, the population is demographically comparable with the Framingham Heart Study cohort that has contributed to voluminous epidemiologic evidence in the cardiovascular field. ^25^ Additionally, some patients and controls moved away from Olmsted County during the study, which limited follow-up duration for these individuals. Furthermore, dividing heart failure into different categories was not possible due to the low number of incidents within each subgroup. Only 13 patients were treated with trastuzumab, so the association of trastuzumab therapy with cardiovascular risk could not be assessed in this study, acknowledging low power to identify associations.

## Conclusions

This case-control study found that patients with a diagnosis of breast cancer or non-Hodgkin or Hodgkin lymphoma treated with anthracyclines had a significantly greater risk of CHF compared with a matched control group in the first year after diagnosis and up to 20 years after cancer diagnosis. Age was another independent risk factor associated with CHF. Further prospective studies are required to balance the potential benefits of anthracycline vs the cardiovascular risks and to develop surveillance models and susceptibility indexes.

## Notes

Supplement 1. **eAppendix.** Diagnostic Codes Used **eFigure 1.** Spline Plot Curve Illustrating CHF Risk Across Different Anthracycline Doses **eFigure 2.** Cumulative Incidence of Congestive Heart Failure for Patients With Cancer Across Time, by Cancer Type

Supplement 2.
Data Sharing Statement
