# Error in Affiliations

**DOI:** 10.1001/jamanetworkopen.2023.4015

**Published:** 2023-03-03

In the Original Investigation titled “Association of Anthracycline With Heart Failure in Patients Treated for Breast Cancer or Lymphoma, 1985-2010,”^1^ published February 3, 2023, there was an error in Dr Goetz’s affiliation, which should have been Division of Medical Oncology, Mayo Clinic, Rochester, Minnesota. This article has been corrected.^1^
