# Supplementary material for: Association of Anthracycline With Heart Failure in Patients Treated for Breast Cancer or Lymphoma, 1985-2010
Source: JAMA Netw Open. 2023 Feb 3;6(2):e2254669. doi: 10.1001/jamanetworkopen.2022.54669 (PMC9898820; doi:10.1001/jamanetworkopen.2022.54669)
Supplement: Supplement 1. — eAppendix. Diagnostic Codes Used eFigure 1. Spline Plot Curve Illustrating CHF Risk Across Different Anthracycline Doses eFigure 2. Cumulative Incidence of Congestive Heart Failure for Patients With Cancer Across Time, by Cancer Type [file jamanetwopen-e2254669-s001.pdf]

Supplemental Online Content

Larsen CM, Garcia Arango M, Dasari H, et al. Association of anthracycline with heart failure in patients treated for breast cancer or lymphoma, 1985-2010. *JAMA Netw Open*. 2023;6(2):e2254669. doi:10.1001/jamanetworkopen.2022.54669

eAppendix. Diagnostic Codes Used

eFigure 1. Spline Plot Curve Illustrating CHF Risk Across Different Anthracycline Doses

eFigure 2. Cumulative Incidence of Congestive Heart Failure for Patients With Cancer Across Time, by Cancer Type

This supplemental material has been provided by the authors to give readers additional information about their work.

eAppendix. Diagnostic Codes Used

80103 (Carcinoma Nos)

81403 (Adenocarcinoma Nos)

84803 (Mucinous Adenocarcinoma)

85002 (Intraductal Carcinoma Noninfiltrating Nos)

85003 (Infiltrating Duct Carcinoma Nos)

85073 (Micropapillary Ca Invasive)

85103 (Medullary Carcinoma Nos)

85133 (Atypical Medullary Carcinoma)

85203 (Lobular Carcinoma Nos)

85213 (Infiltrating Ductular Carcinoma)

85223 (Infiltrating Duct and Lobular Carcinoma)

85233 (Infiltrating Duct Mixed with Other Types Of Carcinoma)

85303 (Inflammatory Carcinoma)

96503 (Hodgkin Lymphoma Nos)

96593 (Hodgkin Lymphoma Nodular Lymphocyte Predominance)

96633 (Hodgkin Lymphoma Nodular Sclerosis Nos)

96643 (Hodgkin Lymphoma Nodular Sclerosis Cellular Phase (Obs 2010+)

96703 (Malignant Lymphoma Small B Lymphocytic Nos (Obs))

96733 (Mantle Cell Lymphoma)

96803 (Malignant Lymphoma Large B-Cell Diffuse Nos)

96843 (Malignant Lymphoma Large B-Cell Diffuse Immunoblastic Nos (Obs 2010+))

96873 (Burkitt Lymphoma Nos)

96903 (Follicular Lymphoma Nos)

96913 (Follicular Lymphoma Grade 2)

96953 (Follicular Lymphoma Grade 1)

© 2023 Larsen CM et al. *JAMA Network Open.*

96983 (Follicular Lymphoma Grade 3)

96993 (Marginal Zone B-Cell Lymphoma Nos)

97023 (Mature T-Cell Lymphoma Nos)

97093 (Cutaneous T-Cell Lymphoma Nos)

97143 (Anaplastic Large Cell Lymphoma T Cell and Null Cell Type)

97273 (Precursor Cell Lymphoblastic Lymphoma Nos)

© 2023 Larsen CM et al. *JAMA Network Open.*

eFigure 1. Spline Plot Curve Illustrating CHF Risk Across Different Anthracycline Doses

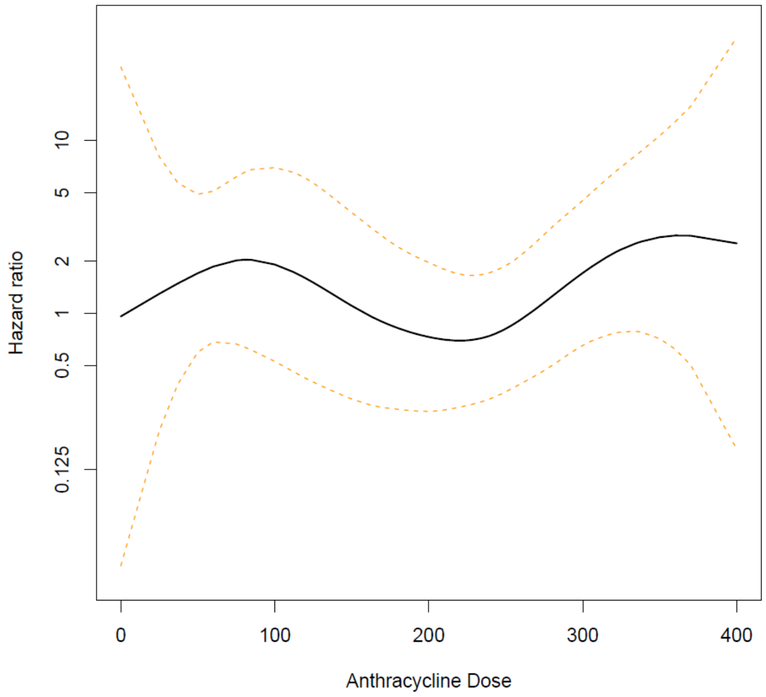

© 2023 Larsen CM et al. *JAMA Network Open.*

eFigure 2. Cumulative Incidence of Congestive Heart Failure for Patients With Cancer Across Time, by Cancer Type

eFigure 2a. Cumulative incidence of congestive heart failure for Breast Cancer patients across time Adjusted by age, sex, Diabetes Mellitus, coronary artery disease, hyperlipidemia, smoking history and obesity HR 3.52 [CI 1.78-6.93] (p <.001)

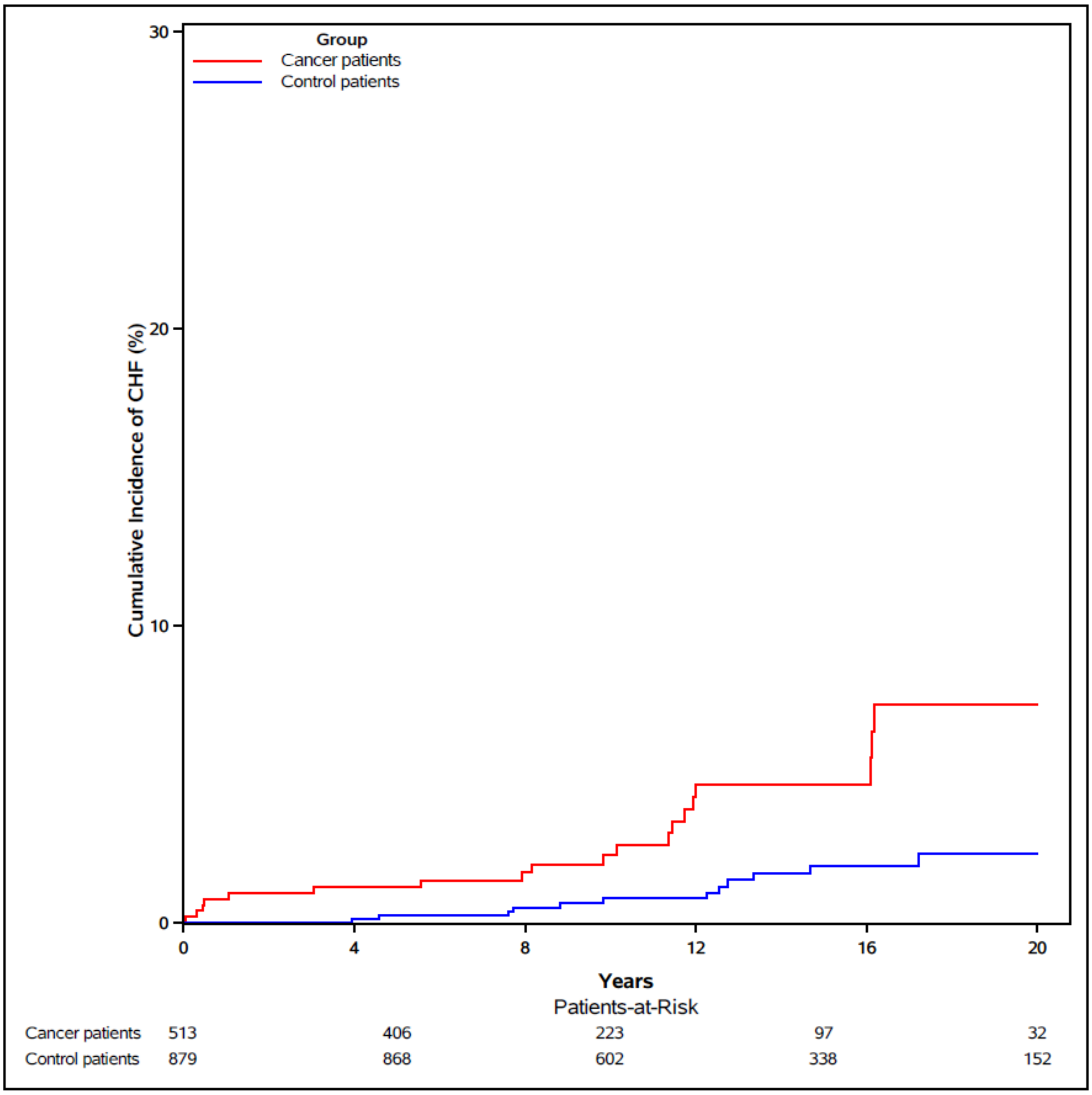

© 2023 Larsen CM et al. *JAMA Network Open.*

eFigure 2b. Cumulative incidence of congestive heart failure for Non-Hodgkin lymphoma patients across time Adjusted by age, sex, Diabetes Mellitus, coronary artery disease, hyperlipidemia, smoking history and obesity HR 1.99 [CI 1.12-3.51] (p =.02)

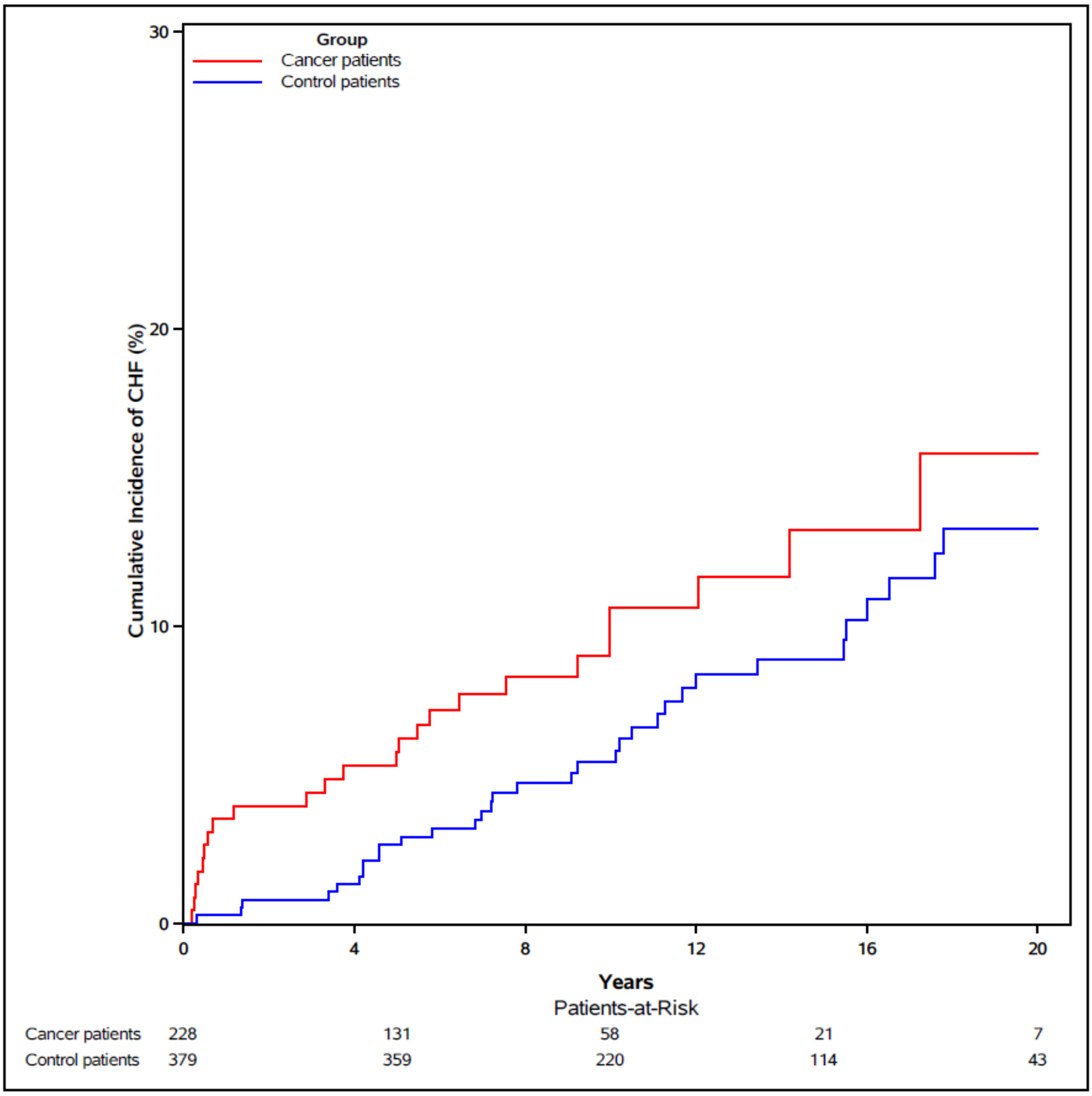

© 2023 Larsen CM et al. *JAMA Network Open.*

eFigure 2c. Cumulative incidence of congestive heart failure for Hodgkin lymphoma patients across time, unadjusted (Only four CHF events)

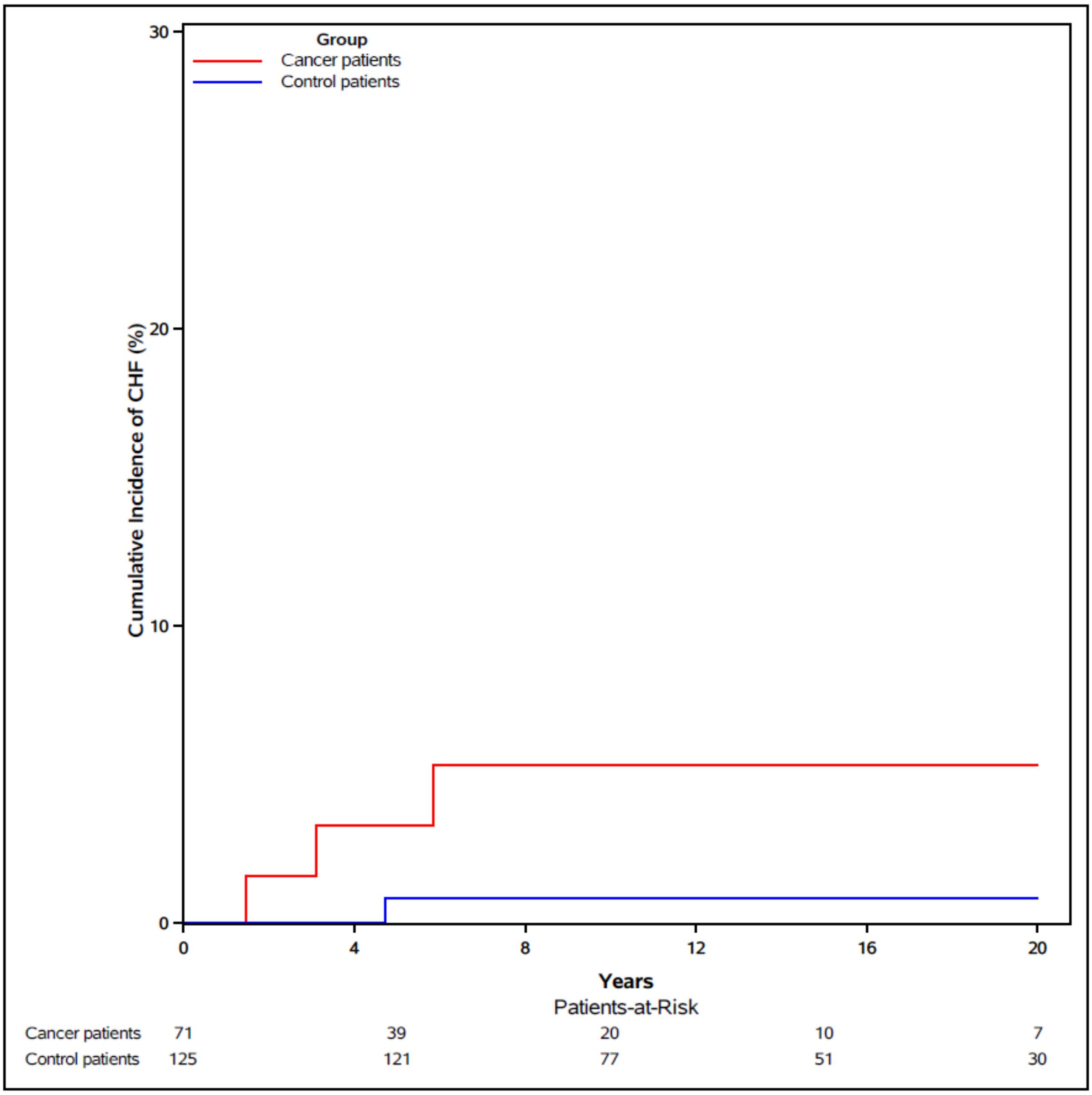

© 2023 Larsen CM et al. *JAMA Network Open.*
