# Supplementary material for: Association of Anthracycline With Heart Failure in Patients Treated for Breast Cancer or Lymphoma, 1985-2010
Source: JAMA Netw Open. 2023 Feb 3;6(2):e2254669. doi: 10.1001/jamanetworkopen.2022.54669 (PMC9898820; doi:10.1001/jamanetworkopen.2022.54669)
Supplement: Supplement 2. — Data Sharing Statement [file jamanetwopen-e2254669-s002.pdf]

Data Sharing Statement

Larsen. Association of Anthracycline With Heart Failure in Patients Treated for Breast Cancer or Lymphoma, 1985-2010. *JAMA Netw Open*. Published February 03, 2023. doi:10.1001/jamanetworkopen.2022.54669

Data
Data available: No
